# In vitro efficacy of synthetic lawsone derivative disinfectant solution on removing dual-species biofilms and effect on acrylic denture surface properties

**DOI:** 10.1038/s41598-023-41531-5

**Published:** 2023-09-08

**Authors:** Jutharat Manuschai, Luelak Lomlim, Pichayaporn Ratti, Jiraporn Kara, Supawadee Naorungroj

**Affiliations:** 1 Department of Conservative Dentistry, Faculty of Dentistry, Prince of Songkla University, Hat Yai, 90112 Songkhla Thailand; 2 Department of Pharmaceutical Chemistry, Faculty of Pharmaceutical Sciences, Prince of Songkla University, Hat Yai, Songkhla 90112 Thailand; 3 Phytomedicine and Pharmaceutical Biotechnology Excellent Center (PPBEC), Faculty of Pharmaceutical Sciences, Prince of Songkla University, Hat Yai, Songkhla 90112 Thailand; 4 Department of Dental Public Health, Sirindhorn College of Public Health Yala, Muang Yala, Yala 95000 Thailand

**Keywords:** Dental biofilms, Pharmaceutics

## Abstract

*Candida albicans* (*C. albicans*) and *Streptococcus mutans* (*S. mutans*) biofilms involve in denture stomatitis. This study compared compound 1 to 2% chlorhexidine gluconate (CHX), Polident, and distilled water (DW) in biofilms reduction and effect on polymethylmethacrylate acrylic (PMMA) properties. The structure of lawsone (naphthoquinone derivative) was modified by the addition of an alkylnyloxy group to yield compound 1. Dual-species biofilms of *C. albicans* and *S. mutans* were developed on PMMA discs. The colony-forming unit count measured the number of residual biofilm cells after exposure to the test agents. PMMA discs were examined for color stability, surface roughness, hardness, and chemical structure after 28 days. At 3 min, compound 1 was less effective than CHX in reducing *C. albicans* (*p* = 0.004) and *S. mutans* (*p* = 0.034) but more effective than Polident in reducing *C. albicans* (*p* = 0.001). At 15 min, no viable cells were detectable for compound 1 and its effectiveness was comparable to CHX (*p* = 0.365). SEM showed fungal cell surface damages in CHX, compound 1 and Polident groups. Only color change was affected by time (*p* < 0.001) and type of test agent (*p* = 0.008), and only CHX reached a clinical perception level. Compound 1 is a promising agent for removing biofilm from the PMMA surface without substantially degrading surface properties.

## Introduction

Denture stomatitis is one of the most prevalent conditions affecting denture wearers^1^. Due to the fact that the surface characteristics of dentures themselves favor bacteria and fungi colonization and serve as a reservoir for pathogenic denture biofilm that can exacerbate oral inflammation, poor denture hygiene is recognized as a significant risk factor for denture stomatitis. Several studies demonstrated a clear correlation between poor denture hygiene and prevalence of denture stomatitis^1–4^. Wearing denture overnight has also been linked to poor denture hygiene and an increased risk of developing denture stomatitis, due to the environment that promotes pathogens overgrowth^1,3^. The prevalence of *Candida* species, in particular *Candida albicans* (*C. albicans*), is accepted as a leading etiological factor in this oral disease^2,3,5^. Thus, effective denture plaque control is one of the most important factors in preventing and treating the disease.

The most common method for cleaning dentures is a regular brushing with soap and water. However, brushing dentures alone is insufficient to eliminate biofilm^6–8^. In addition, the majority of denture wearers are elderly, making it difficult to effectively clean their dentures^1^. Improperly cleaned denture rapidly accumulate pathogenic denture biofims^3^. Previous studies showed that immersing dentures in disinfectant solutions containing chemical agents are effective method for reducing the number of contaminating organisms^6,9^. Several disinfectant solutions have been suggested for denture disinfection^7,10^. Alkaline peroxides are the most commonly used denture cleanser; however, their efficacy in biofilm removal is conflicting^6,9,11^. A previous study showed that immersing acrylic dentures in alkaline peroxide for the time specified in the manufacturer’s instruction was insufficient for their decontamination^11^, while several studies suggested that these solutions may be useful when combined with a mechanical method^6,12^. Moreover, some studies showed that prolonged use of alkaline peroxide affected the physical properties of acrylic denture^13,14^. Chlorhexidine gluconate (CHX) is a broad-spectrum antimicrobial agent, which is a commonly used antiseptic substance in dental therapy^15^. Currently, it is also employed as a disinfectant agent for cleaning non-living clinical surfaces. The approved concentration of active CHX ranges from 0.06% up to 2%, with the 2% concentration serving as an overnight denture disinfectant^15,16^. Several studies suggested that CHX is effective on denture biofilm removal^16–18^. However, CHX is not to be recommended for denture disinfection due to the concurrent staining associated with prolonged use^7,15,19^. Denture disinfectant solutions should be effective without compromising the material properties or clinical longevity of the dentures.

Lawsone is a member of the naphthoquinone (NQ) class of natural products found in the leaves of the henna plant (*Lawsonia inermis*). Lawsone and its derivatives have shown a variety of bioactivities including antifungal and antibacterial activities^20,21^. Our previous study synthesized new lipophilic lawsone derivatives with enhanced antifungal and antibacterial activities for dental applications by changing the 2-hydroxyl group of lawsone to different alkynyloxy groups. Among the compounds in the series, 2-(prop-2-ynyloxy)naphthalene-1,4-dione (compound 1) demonstrated promising anti-candida, anti-cariogenic bacteria activities, and *Streptococcus mutans* (*S. mutans*) biofilm inhibition properties^22,23^. Compound 1 was efficiently synthesized via a simple alkylation reaction of lawsone with propargylbromide under basic condition and obtained as a yellow solid, with molecular weight of 212.20 Dalton, and melting point of 149–151 °C. A previous study reported that spraying acrylic discs with an antifungal spray containing compound 1 reduced number of viable *C. albicans* single-species biofilm more effectively than immersion in Polident^22^. Although most attention in denture stomatitis focus on the *Candida* component of the biofilm, bacterial species such as *Staphylococcus* and *Streptococcus* have been found to coexist with *Candida* during denture infection^24–26^. Several evidence showed that *C. albicans* become more invasive, exacerbating mucosal tissue infection and destruction, when coinfected with oral streptococci^27–29^. *S. mutans* is highly detected on the denture surface of denture stomatitis patients^25^. It has been demonstrated that strategies used to control single-species biofilms had little effect on dual-species biofilm of *S. mutans* and *C. albicans* due to synergistic interaction^30^. Furthermore, exopolysaccharides produced by *S. mutans* enhanced antifungal drug tolerance in dual-species biofilm^31^. To maintain the efficacy of disinfectant solution against complex biofilms, the exposure time may need to be modified. The formation of more complex biofilm structure, a dual-species biofilm of *C. albicans* and *S. mutans* associated with denture stomatitis, is more appropriate for evaluating the in vitro efficacy of denture disinfectant solution. The present study therefore aimed to (i) evaluate the efficacy of compound 1 disinfectant solution on the removal of dual-species denture biofilm constituted of *C. albicans* and *S. mutans* with short and long exposure times; and (ii) examine the effect of prolonged use of this disinfectant solution on the physical and chemical properties of acrylic dentures.

The null hypotheses of this study were that there was no difference between compound 1 and the other test agents in terms of (i) their effect on the biofilm viability, and (ii) physical properties of acrylic specimens (color, surface roughness, surface hardness) after 28 days of immersion.

## Results

### Viability of residual biofilm-forming cells and morphological observations

**Figure 1:**
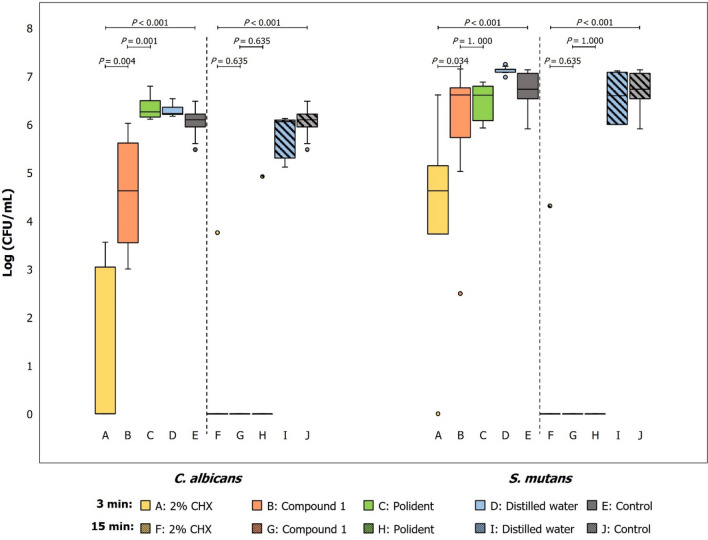
The distribution of viable cell count (Log CFU/mL) of *C. albicans* and *S. mutans* in the dual-species biofilms formed on PMMA discs after exposure to test agents for 3 and 15 min, and the untreated control.

In the untreated biofilms, median values of log (CFU/mL) revealed a higher amount of *S. mutans* compared with *C. albicans* (Supplementary Table 1). For 3-min contact time, both species of biofilm cells were still alive in all groups. The number of *C. albicans* cells in the compound 1 groups was significantly lower than the Polident group (*p* < 0.001). However, there was no difference in the number of *S. mutans* cells between both groups (*p* = 1.000), when exposed to the test agents for 3 min. For CHX, compound 1 and Polident groups, the number of viable cells was reduced in a contact time-dependent manner, which were statistically lower than those in the untreated control group (*p* < 0.001). In compound 1 group, there were no detectable viable cells in both species after immersed for 15 min (Fig. 1). Immersion in DW neither 3 nor 15 min reduced *C. albicans* and *S. mutans* biofilms (*p* > 0.050).

**Figure 2:**
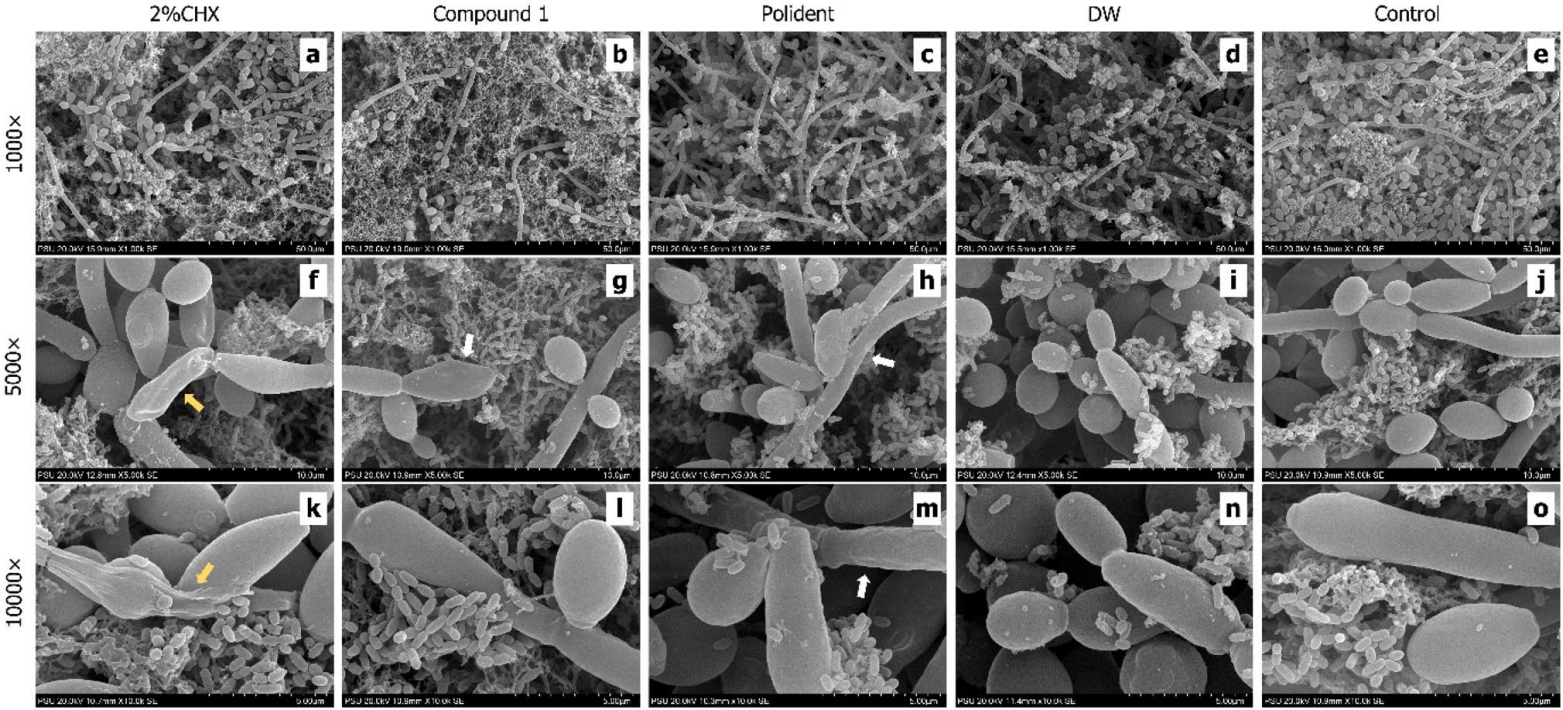
Representative SEM images of dual-species biofilm structure of *C. albicans* and *S. mutans* remaining on the PMMA discs after exposure to each test agent (2% CHX: **a**, **f**, **k**; Compound 1: **b**, **g**, **l**; Polident: **c**, **h**, **m**; DW: **d**, **i**,** n**; Control: **e**,** j**, **o**) for 15 min and untreated control. Yellow arrows: fungal cell surface damage in the CHX group; White arrows: slightly wrinkle fungal cells in compound 1 and Polident groups.

Morphological observation of dual-species biofilm under scanning electron microscope (SEM) revealed abundant *C. albicans* cells (both yeast and hyphal forms) were surrounded by cluster of *S. mutans* cells which embedded in extracellular polymeric substance (EPS) interspersed with open water channels. Some of the *S. mutans* clusters attached to the surface of fungal cells (Fig. 2f–j). Yeast cells were observed in the CHX (Fig. 2a) and compound 1 (Fig. 2b) groups less than the others (Fig. 2c–e). Under 5000 × and 10,000 × magnifications, this study detected fungal cell surface damage in the CHX group (Fig. 2f,k). Whereas, compound 1 and Polident groups presented slightly wrinkle in some fungal cells (Fig. 2g,h), however this was not distinctly different from the fungal cell morphology of the DW and untreated control groups (Fig. 2i,j). When compared with the untreated control (Fig. 2o), there was not noticeable change in *S. mutans* cell morphology after immersion in test agents (Fig. 2k–n).

### Physical and chemical properties of acrylic denture

#### Color stability

**Figure 3:**
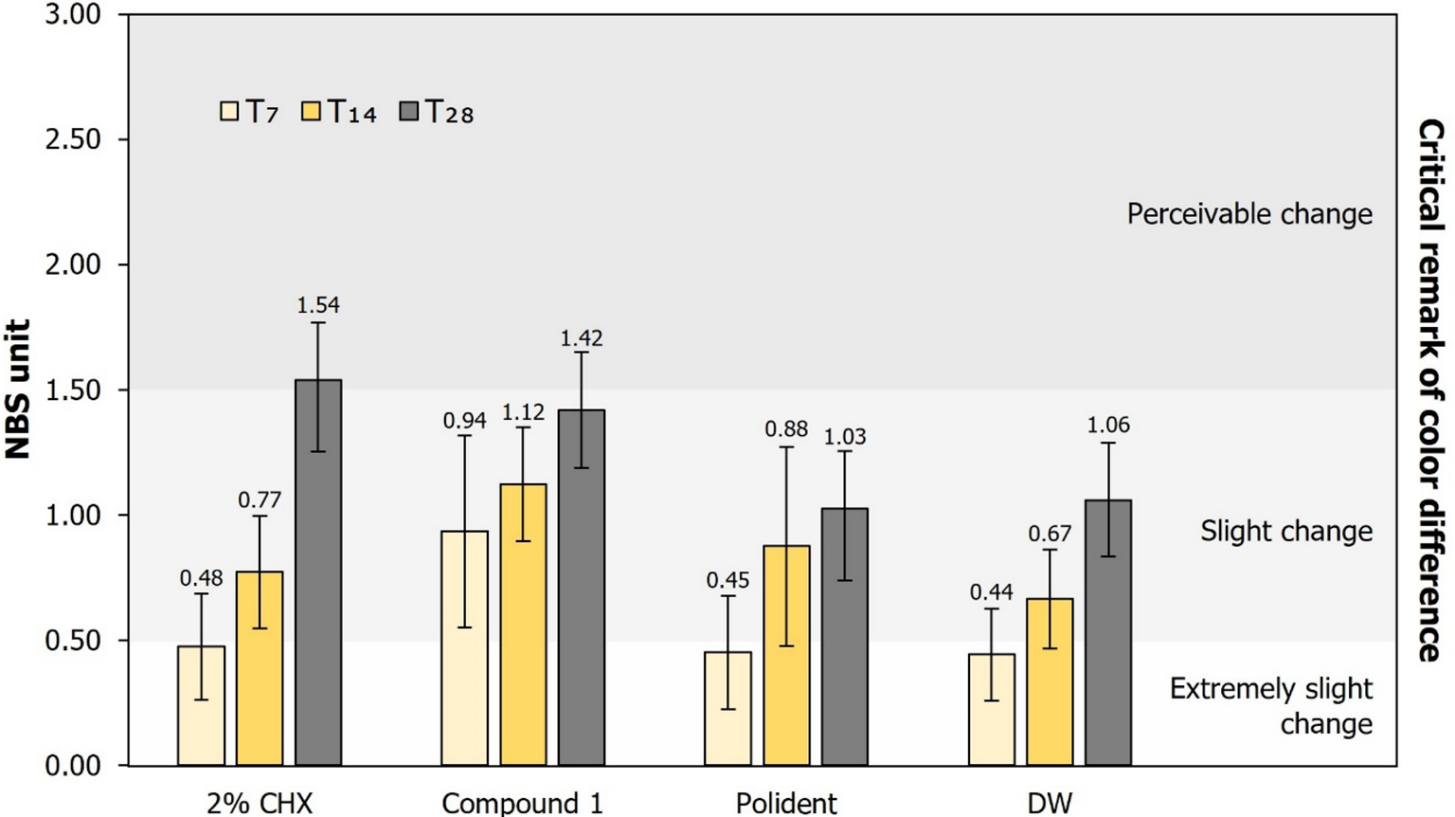
Mean and standard deviation of National Bureau of Standard (NBS) units and critical remarks of the color difference of PMMA discs after immersion in each test agent for 7, 14 and 28 days.

When specimens were immersed in each test agent for 28 days, it was observed that the total color change (ΔE) mean value of the CHX group was the greatest (1.67 ± 0.31), followed by the compound 1 (1.54 ± 0.25), Polident (1.12 ± 0.31) and DW (1.15 ± 0.35) groups respectively. Figure 3 showed that all groups underwent color change over time. There was only the color change of CHX group which be perceptible after immersed for 28 days. According to the results of statistical analysis, the type of test agents (*p* = 0.008) and immersion time (*p* < 0.001) had a statistically significant effect (*p* = 0.018) on color stability of acrylic denture base. Statistically significant differences for color change occurred between the CHX and DW (control) groups (*p* = 0.019) after immersion for 28 days.

#### Surface roughness and surface morphology

**Figure 4:**
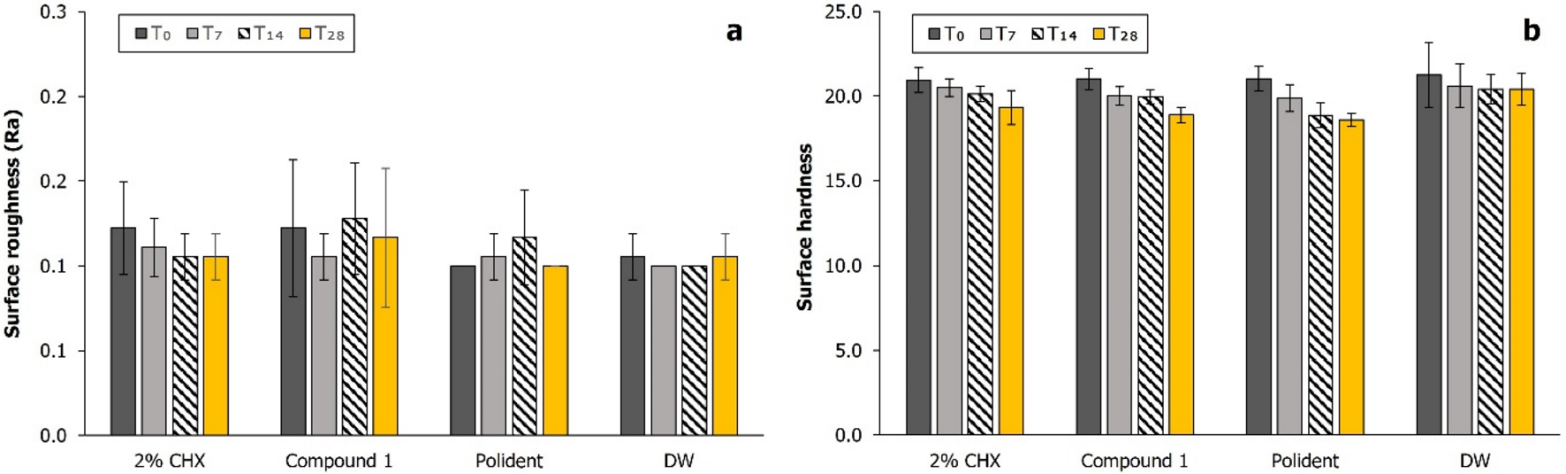
Mean and standard deviation of (**a**) surface roughness and (**b)** surface hardness values of PMMA discs after immersion in each test agent for 0, 7, 14, and 28 days.

**Figure 5:**
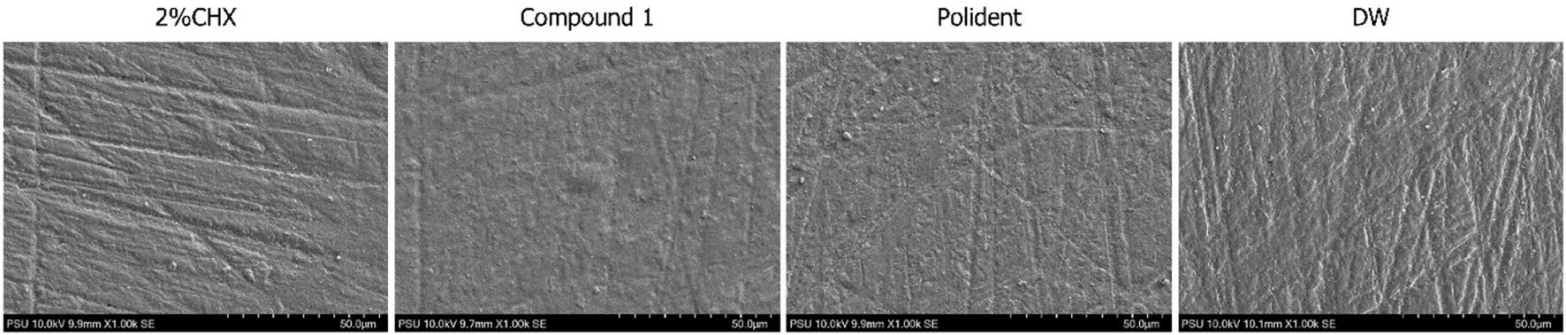
The surface morphology of PMMA discs after immersion in each test agents for 28 days.

The results showed that the type of test agents (*p* = 0.217) and immersion time (*p* = 0.475) did not affect roughness of acrylic denture base. There was no statistically significant difference in the mean of average roughness (Ra) values among all groups (*p* = 0.243) (Fig. 4a). Changes on surface morphology in each group were further analyzed by SEM (Fig. 5). There was no obvious difference in surface morphology between specimens immersed in compound 1 disinfectant solution and DW (control).

#### Surface hardness

For all groups, mean Vickers hardness values (HV) decreased from 21.25 to 20.93 kg/mm^2^ at baseline to 20.41–18.59 kg/mm^2^ after 28 days of immersion (Fig. 4b). The results of statistical analysis showed that immersion time had a statistically significant effect on the surface hardness of acrylic denture base (*p* < 0.001), whereas the type of test agents had not a statistically significant effect (*p* = 0.050).

#### Determination of chemical change on acrylic denture base

**Figure 6:**
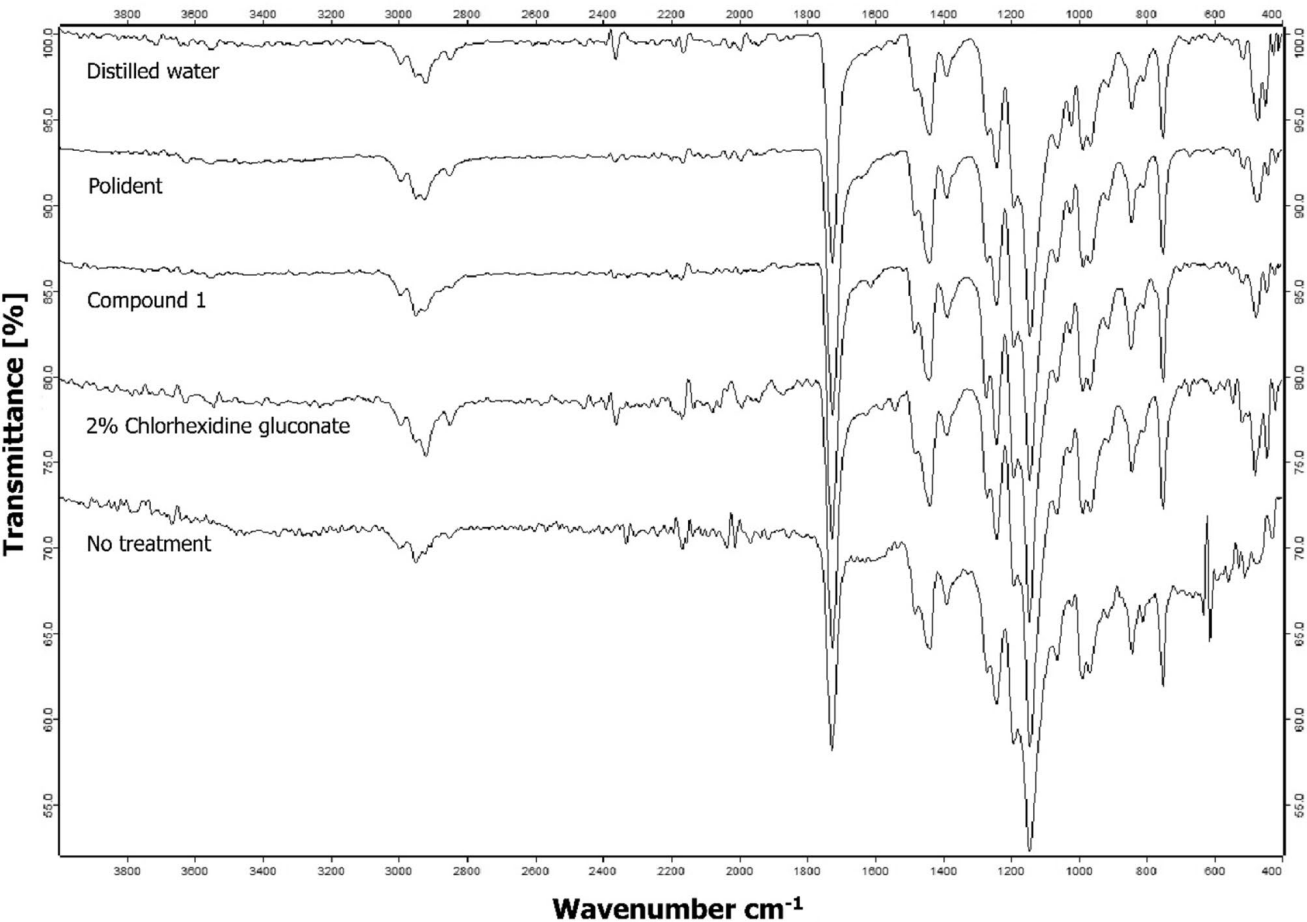
FTIR spectrum of the PMMA discs after immersion in 2% chlorhexidine gluconate, compound 1, Polident, and distilled water for 28 days, compared with no treatment disc.

When compared the infrared spectra of PMMA discs immersed in compound 1 disinfectant solution for 28 days with the one immersed in DW, no change of absorption band has been observed (Fig. 6). That means compound 1 did not chemically affect the composition of PMMA material.

## Discussion

Denture disinfection solutions must have the ability to properly remove biofilm while having no detrimental effects on acrylic dentures. Within this study, effectiveness of compound 1 depended on contact time and microorganisms. There was a significant reduction of biofilm viability of compound 1, CHX, and Polident compared to DW and untreated control groups. Our first null hypothesis was thus rejected. In addition, we found no significant difference in color, surface roughness, and surface hardness of PMMA between compound 1 and the other test agents. Consequently, the second null hypothesis failed to be rejected.

Compound 1 is a synthetic derivative of NQs in which the 2-hydroxyl functionality has been replaced with an O-alkynyl group. It was anticipated that increasing the lipophilicity with this modification would enhance cell membrane permeation, thereby increasing antimicrobial effects on cells^32^. Compound 1 has antifungal and antibacterial properties^22,23^. Moreover, this NQs derivative exhibited potential antibiofilm activities against *S. mutans* and *C. albicans* single-species biofilms, according to previous reports^22,23^. The antimicrobial and antibiofilm activities of this compound may be attributable to its abilities to generate reactive oxygen species that can affect biomolecules such as DNA, protein and lipid, resulting in intracellular damage and apoptosis^21^. However, in this study, the SEM images do not reveal obvious cell damage; it is likely that the target sites were intracellular organelles. Further investigation using transmission electron microscope (TEM) imaging technique may be beneficial to illustrate changes at intracellular level, such as mitochondrial damage, cell membrane damage, and cytoplasmic abnormalities, caused by compound 1.

Based on a previous study^22^, the 400 µg/mL concentration of the compound 1 disinfectant solution was the most effective on eliminate single-species biofilms of *C. albicans* from acrylic denture. However, spraying with the compound 1 (400 µg/mL) disinfectant solution in the present study for 3 min disrupted but did not completely eliminate the dual-species biofilms, similar to immersion in 2% CHX for 3 min. When the immersion time was increased to 15 min, compound 1 maintained antimicrobial activity in dual-species biofilms with comparable efficacy against single-species biofilms of *C. albicans*. The dual-species biofilm was more complex, structured and organized in its extracellular matrix, making both species more resistant to environment stresses^33^. In this study, biofilm communities exhibited a highly organized ecosystem characterized by dispersed water channels. The biofilm cells were embedded in an EPS matrix, which was responsible for intercellular interactions and cells protection from hostile environment^34^. Previous studies have demonstrated that *C. albicans* and *S. mutans* cells exhibit synergistic activity when cultured together^33,35^. The presence of *C. albicans* in a biofilm modifies the physical environment, promoting the increase of exopolysaccharides and consequently, accumulation and formation of microcolonies by *S. mutans*. Additionally, *C. albicans* can secrete its own matrix products, including β-glucan^33^. Therefore, our study suggests that the complete elimination of mixed-species biofilm can be accomplished by extending the contact time beyond that of single-species biofilms.

Spraying compound 1 with 3-min contact time was more effective in reducing biofilm viability than immersion in a commercial product Polident, an alkaline peroxide-type denture cleanser, for 3 min according to the manufacturer’s instructions. The number of viable biofilm cells in specimens immersed in Polident for 3 min was comparable to immersion in DW or no treatment, according to our study. This is in agreement with a previous finding^11^ that a short immersion time (< 15 min) in an alkaline peroxide-based denture cleanser dosed not result in complete disinfection. Moreover, a previous study suggested that alkaline peroxide-type denture cleansers are complementary to denture hygiene and must be used in conjunction with mechanical methods for more effective biofilm elimination^11^. The Polident usage instructions recommend brushing a denture with its solution after soaking for 3–5 min. Therefore, it is anticipated that Polident will have a more consistent effect on biofilm removal when the contact time is prolonged or when combined with mechanical methods, such as brushing.

Despite immersion in CHX has been shown to be the most effective in removing biofilm from denture, which consistent with the previous studies^16–18^. Continuous exposure to a high concentration of CHX may induce shifts in microbiota composition. In addition, one of the primary disadvantages of CHX solutions used in dentistry is that they may cause extrinsic staining of dental materials. Previous studies have documented the impact of CHX disinfectant on the color and mechanical properties of acrylic dentures^36,37^.

In our study, 28-days immersion simulated daily 15-min denture treatment for 7 years. Although this simulation was continuous immersion and did not include factors such as thermal cycling and masticatory force that affect surface condition, the results of this investigation indicate a tendency toward long-term use of denture disinfectant. The physical and chemical properties of acrylic denture bases immersed in compound 1 disinfectant solution presented the same behaviors of those immersion in DW. Even when immersed in DW, the acrylic denture bases undergo color change over time. Similar results were reported in previous studies^36,38–40^. The color change could be the result of liquid absorbance. When water molecules are absorbed by the resin, they function as plasticizers, resulting in linkage cleavage, component dissolution and intrinsic pigment degradation^38,41^. In this study, the specimens immersed in 2% CHX exhibited the most significant color change when compared to the DW group. This is a result of the local precipitation reaction between the cationic CHX molecule and acrylic material^42^. PMMA materials immersed in a disinfectant solution may experience a decrease in surface hardness due to monomer dissolution as time passes. In agreement with previous studies^37,43^, our study showed that surface hardness of each test group decreased as immersion time increased. Nonetheless, some studies have reported an increase in surface hardness after 21–28 days of immersion in disinfectant solutions^38,44^. The results were explained by the release of residual monomers from the polymeric materials, contributing to the increase of hardness values.

This study demonstrated that the surface roughness did not change after immersion in any of the test agents. This result is consistent with the previous studies^14,38,39^. The stability of heat-cured acrylic resin may be attributed to its reticulated polymeric structure, which results from a thermal polymeric reaction in which a high rate of monomers converts into polymers, thereby making the material more stable^45^. However, some studies have reported surface roughness changes following alkaline peroxide cleanser immersion^43,46^.

In addition to the ability to effectively eliminate denture biofilm and compatibility with denture properties, the absence of toxicity and irritation to oral epithelial cell is a characteristic of a desirable denture disinfectant. The amount of residual compound remaining on the denture surface after cleaning should be as least as possible. Therefore, additional experiment was conducted in our study to ascertain the quantity of residual compound 1 in PMMA samples after 15 min of immersion and two 10-s rinses. On the disc surfaces, about 70% of compound 1 was detected (data not shown). In order to ensure that the compound 1 disinfectant solution is safe for clinical use, it is suggested that longer rinsing times and irritation testing be performed.

This study has both strength and limitations. Despite the favorable results of the efficacy of the new synthetic compound 1 disinfectant solution to serve as an alternative agent for denture disinfection in the future, it should be interpreted with care as the study was an in vitro work and cannot completely imitate the complex natural biofilms. In order to simulate the in vitro biofilm model as closely as possible to the natural denture biofilm, the dual-species biofilms containing fungal and bacterial pathogens that strongly associated to denture stomatitis was used in our study^1,25,26^. However, non-albicans species or other oral bacterial species were also identified in denture biofilm^3,5,24^. Thus, the susceptibility of multi-species to compound 1 may be differ. Furthermore, using acrylic discs does not account for the complex topology of the denture structure. Further work needs to be performed to validate the efficacy of compound 1 disinfectant solution in clinical practice, as well as the long-term impacts on surface and chemical properties of acrylic dentures in laboratory setting under thermal cycling.

## Conclusion

This in vitro study demonstrated that:

1. Compound 1, CHX, and Polident exhibited comparable efficacy in disinfecting *C. albicans* and *S. mutans* biofilms on PMMA surface, when immersed for 15 min. However, it should be noted that none of these agents were able to completely remove the biofilms according the SEM.
2. With the exception of a color change that reached a clinical perceptible level after 28 days of immersion in CHX, PMMA’ s physical characteristics, including surface roughness, hardness, and color were slightly altered following exposure to all chemical agents.

## Methods

### Preparation of denture disinfectant containing 400 µg/mL of compound 1

**Figure 7:**
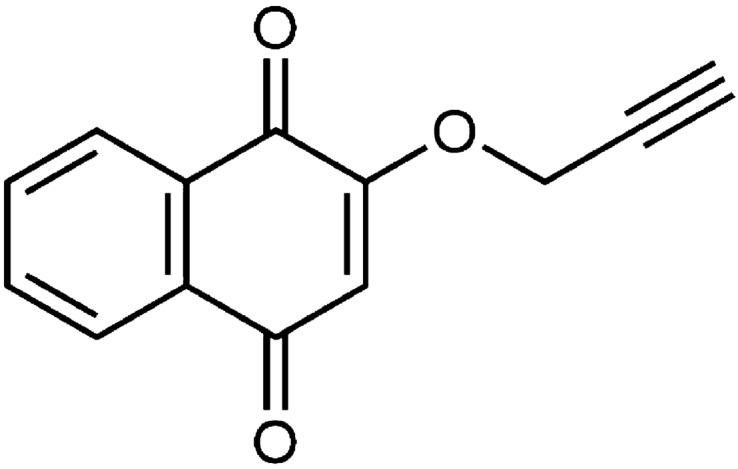
The chemical structure of 2-(prop-2-ynyloxy)naphthalene-1,4-dione (compound 1).

Compound 1 (Fig. 7) was synthesized, purified, characterized, and prepared as a denture disinfectant according to the previous protocol^22^. Briefly, compound 1 (20 mg), poloxamer 407 (1 g) (P.C. Drug Center, Bangkok, Thailand), saccharin (0.05 g) (Vidhyasom, Bangkok, Thailand), and menthol (0.025 g) (Vidhyasom, Bangkok, Thailand) were mixed in a mortar. Glycerin (5 mL) (P.C. Drug Center, Bangkok, Thailand) was gradually added, and the mixture was grinded until it became a smooth paste. Distilled water (25 mL), ethanol (12.5 mL) (P.C. Drug Center, Bangkok, Thailand), and paraben concentrated (0.5 mL) (P.C. Drug Center, Bangkok, Thailand) were added and mixed thoroughly. After the addition of peppermint oil (1 drop) (Vidhyasom, Bangkok, Thailand), the solution was transferred to a cylinder and the volume adjusted to 50 mL with distilled water. The denture disinfectant solution was obtained as a clear solution (pH 7.32). The solution was freshly prepared and stored in an amber glass bottle. The content of compound 1 ranged from 96.64 to 101.66% labeled amount as measured by high-performance liquid chromatography described in our previous work^22^.

**Figure 8:**
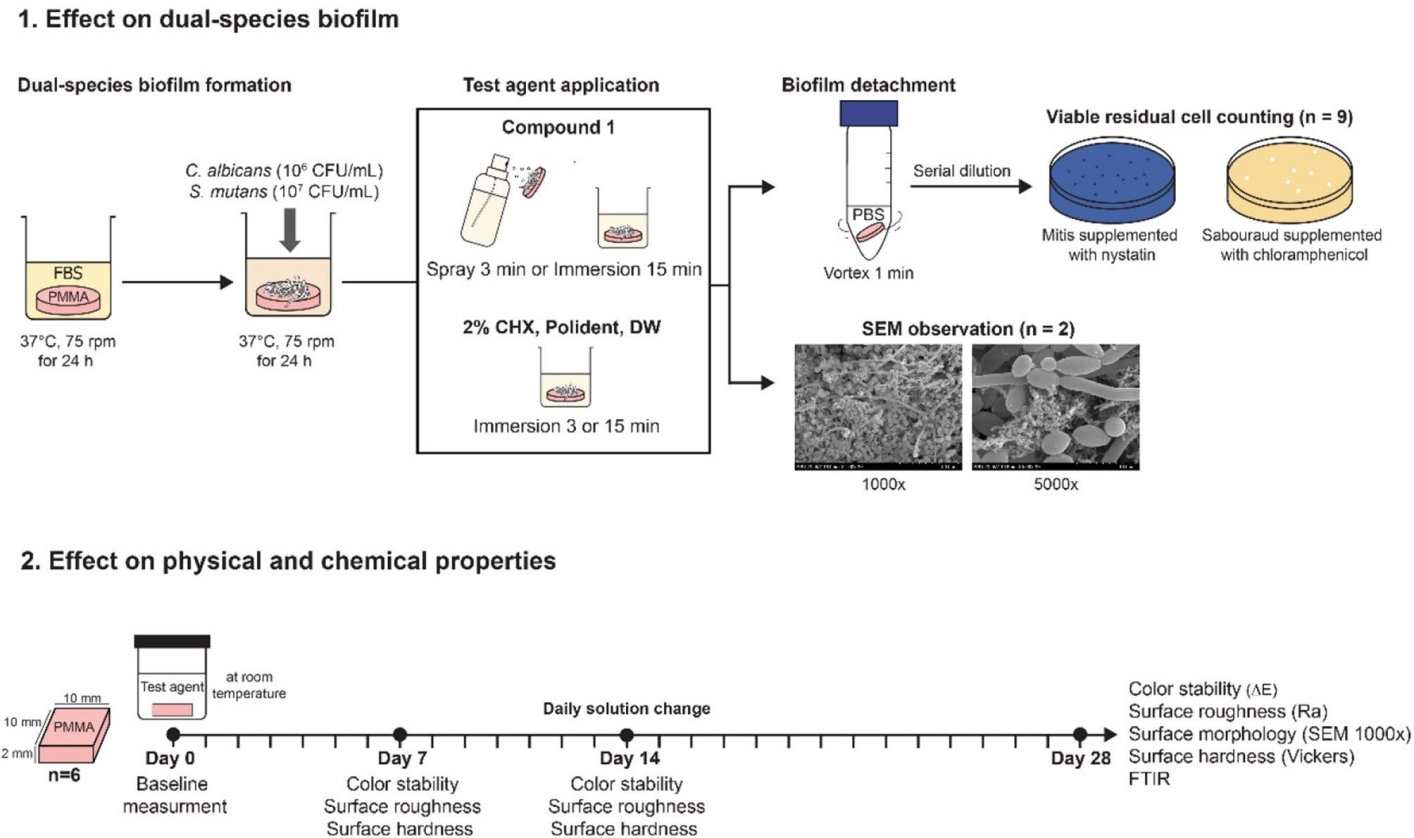
Experimental flow chart.

The denture disinfectant containing compound 1 was evaluated in the aspects of (i) the efficacy in removing biofilms produced by *C. albicans* and *S. mutans* from the acrylic denture surfaces, and (ii) the effect of this solution on the physical and chemical properties of acrylic denture base as illustrated in Fig. 8.

### Specimen preparation

For biofilm assay, the acrylic discs were prepared as described previously^22^. Briefly, heat-cured acrylic resin (Vertex Rapid Simplified; Vertex-Dental, Zeist, Netherlands) was mixed according to the manufacturer’s instructions, and the mixtures were packed in a mold with a cylindrical shape (diameter 10 mm × 5 cm). After polymerization, a cylinder-shaped acrylic resin was cut using a low-speed diamond saw (Isomet 1000; Buchler, Lake bluff, IL, USA) to obtain a mean (± SD) thickness of a disc-shaped specimen of 2.0 (± 0.1) mm. All specimen surfaces were then polished with 600-grit abrasive paper under water to obtain a surface roughness between 0.3 and 0.4 µm, simulating a denture fitting surface^29^. All the specimens were sterilized with hydrogen peroxide gas. The total of 99 disc-shaped specimens were randomly assigned to the untreated control group and 8 test groups according to 4 test agents and 2 contact times (n = 11 per group; 9 samples for viable cell count; 2 samples for morphological observation). Test agents were 2% CHX, compound 1 (400 µg/mL), Polident (Stafford-Miller Ireland, Waterford, Ireland), and DW. The contact times were 3 and 15 min. The number of samples for viable cell count were defined by performing in triplicate in 3 independent experiments (Fig. 8).

For physical properties evaluation, rectangular (10 × 10 × 2 mm^3^) heat-cured acrylic bases were fabricated using compression molding technique, subsequently polymerized and reduced residual monomers according to the above-mentioned protocol. All specimen surfaces were then polished with 400, 600, 1000, and 1500-grit abrasive papers under water to obtain a surface roughness between 0.1 and 0.2 µm, simulating a polished surface of denture^47^. The sample size was defined considering an effect size of 2, 0.05 level of significance and 80% power. The total of 24 specimens were randomly assigned to 4 groups (n = 6) according to test agents as mentioned above, using DW group as a control.

### Strains and culture conditions

*C. albicans* (DMST 5185) and *S. mutans* (DMST 41283) were used in this study. *C. albicans* frozen stocks was subcultured onto Sabouraud Dextrose Agar (SD) (HIMEDIA; HiMedia Laboratories, Mumbai, India) and incubated at 37 °C for 24 h. *S. mutans* frozen stock was subcultured onto brain heart infusion agar (BHI) (HIMEDIA; HiMedia Laboratories, Mumbai, India) and incubated with 5% CO_2_ at 37 °C for 48 h.

To prepare *C. albicans* and *S. mutans* cell suspensions for biofilm assay, a loopful of the agar stock cultures was transferred to SD broth supplemented with 50 mM glucose and BHI broth respectively, overnight cultured at 37 °C for 24 h. *S. mutans* was incubated in the condition with 5% CO_2_. Then, each overnight culture (1 mL) was inoculated into fresh media (9 mL), *C. albicans* was incubated at 37 °C for 18 h (mid-log phase), while *S. mutans* was incubated with 5% CO_2_ at 37 °C for 6 h (mid-log phase). Each resultant culture was centrifuged at 4000 rpm at 4 °C for 20 min, and supernatant was discarded. The cell pellets were washed twice with 0.1 M phosphate-buffered saline solution (PBS; pH 7.2–7.3). *C. albicans* and *S. mutans* cell pellets were re-suspended in tryptic soy broth (TSB) (Difco; Becton Dickinson, Sparks, MD, USA) supplemented with 5% glucose and BHI supplemented with 5% sucrose respectively. *C. albicans* and *S. mutans* cell suspensions were standardized to 10^6^ and 10^7^ CFU/mL respectively, with a later colony count by drop plate method.

### Biofilm assay

Acrylic discs were incubated with fetal bovine serum (FBS) (Gibco; Life Technologies, Carlsbad, CA, USA) in a shaker incubator (Excella E24 Incubator Shaker Series; New Brunswick Scientific, Enfield, CT, USA) at 75 rpm and 37 °C for 24 h. Then, specimens were washed once with 0.1 M PBS, and placed in 24-well plate. Dual-species biofilms were developed by adding 500 µL of each cell suspension in a well of 24-well plate. The plates were incubated aerobically in a shaker incubator at 75 rpm and 37 °C for 24 h.

After dual-species biofilm growth for 24 h, each biofilm sample was gently washed with 1000 µL of 0.1 M PBS twice for 10 s each time to remove any non-adherent cells. The samples without further exposed to test agents were used as the untreated control. For the other groups, biofilm samples were immersed in each test agent as mentioned above for 3 and 15 min, except 3 min-Compound 1 group was treated by spraying according to a previous study^22^. Following exposure to test agents, residual biofilm samples were washed twice with 1000 µL of 0.1 M PBS.

### Viability of residual biofilm-forming cells

Viable residual cell counting was performed as described previously, with slight modifications^22^. Briefly, the biofilm sample was vortexed for 1 min to detach the biofilm from the acrylic surface. The resultant suspension was serially diluted and dropped (20 µL × 5 drops/each dilution) on SD agar supplemented with chloramphenicol (10 µg/mL) and Mitis agar supplemented with nystatin (250 units/mL) for *C. albicans* and *S. mutans*, respectively. Fungal CFU counting was performed after aerobic incubation at 37 °C for 24 h, while bacterial CFU counting was performed after incubation with 5% CO_2_ at 37 °C for 48 h.

### Morphological observation using SEM

Two residual biofilm samples per group were subjected to SEM observation. The samples were prepared as previously described^22^, and observed using a SEM (Hitachi SU3900; Hitachi High-Tech, Tokyo, Japan) at 1000 × and 5000 × magnifications.

### Physical and chemical properties of acrylic denture

The effect of denture disinfectant solutions on color stability, surface roughness, surface hardness and chemical change of acrylic denture base were analyzed by overnight immersion in each test agent for 28 days at room temperature, with the test agents being changed daily. The physical properties were evaluated at 0, 7 14, and 28-day immersion, and the determination of chemical change was performed after 28-day immersion.

#### Color stability

The color of specimens was measured with a spectrophotometer (ColorQuest XE; Hunter Associates Laboratory, Reston, VA, USA) using the Commission Internationale de l'Eclairage (CIE) L*a*b* system^48^. All measurements were made against a black background and repeated in triplicate. Mean values of three-color coordinates were recorded. The ΔE of each specimen was calculated using the formula:

$$\Delta {\text{E }} = \, [(\Delta {\text{L}}*)^{2} + (\Delta {\text{a}}*)^{2} + (\Delta {\text{b}}*)^{2} ]^{1/2} .$$

The ΔE were converted to National Bureau of Standards (NBS) unit using the formula: NBS units = ΔE × 0.92, and correlated with critical remarks of color difference (Table 1).

**Table 1:** National Bureau of Standards (NBS) units of color difference^38^.

NBS units	Critical remarks of color difference
0.0–0.5	Extremely slight change
0.5–1.5	Slight change
1.5–3.0	Perceivable change
3.0–6.0	Marked change
6.0–12.0	Extremely marked change
12.0 or more	Change to another color
Colorimetry National Bureau of Standards Monograph 104; 1968: 47	

#### Surface roughness and surface morphology

The surface roughness (Ra, µm) was measured using a profilometer (Surfcorder SE2300; Kosaka Laboratory, Tokyo, Japan). For each specimen, three measurements about 0.5 mm apart were performed 5.0 mm in length at the center of the specimen, with the cutoff length of 0.8 mm at a stylus speed of 0.5 mm/s. Mean Ra value was calculated for statistical analysis. For surface morphology analysis, one specimen per group was randomly selected, gold sputtered-coated and then observed using SEM (Hitachi SU3900; Hitachi High-Tech, Tokyo, Japan) at 1000 × magnification.

#### Surface hardness

The surface hardness of specimens was measured using a microhardness tester (Mitutoyo HM-211; Mitutoyo, Kanagawa, Japan). Three indentations were made for each specimen with Vickers diamond indenter^49^ under a load of 10 g for 15 s, and mean HV (kg/mm^2^) was calculated for statistical analysis.

#### Determination of chemical change on acrylic denture base

One specimen from each group was chosen as a representative, and a specimen without treatment served as the PMMA standard. The specimens were washed in an ultrasonic cleanser for 15 min before being dried. The chemical change on the acrylic denture base after immersion with compound 1 formulation and other test agents was analyzed from attenuated total reflectance-Fourier transform infrared spectroscopy (ATR-FTIR) at 25 °C using the FTIR (VERTEX 70; Bruker, Ettlingen, Germany). The spectra were collected in the range between 4000 and 400 nm from 64 scans at a resolution of 4 cm^-1^.

### Statistical analysis

The statistical analysis was performed using STATA version 16.1 (StataCorp, College Station, TX, USA). Median, minimum and maximum of log (CFU/mL) for biofilm viability were reported. The distribution of data was illustrated by box plot and bar graph. Statistical significance was set at 0.05. The normality of data was evaluated by the Shapiro–Wilk test, and the homogeneity of variance was tested using the Levene statistical test. The efficacy of the denture cleansers in removing dual-species biofilm was compared by the Mann–Whitney *U* test with Bonferroni correction for multiple comparison. The effect of denture cleanser type and immersion time on the physical properties of acrylic denture was tested by the repeated measures ANOVA, followed by the Bonferroni post-hoc test.

### Supplementary Information

Supplementary Table 1.

## Data Availability

The dataset used and/or analyzed in this study are available from the corresponding author on reasonable request.
