# Supplementary material for: In vitro efficacy of synthetic lawsone derivative disinfectant solution on removing dual-species biofilms and effect on acrylic denture surface properties
Source: Sci Rep. 2023 Sep 8;13:14832. doi: 10.1038/s41598-023-41531-5 (PMC10491685; doi:10.1038/s41598-023-41531-5)
Supplement: Supplementary file 1 — Supplementary Table 1. [file 41598_2023_41531_MOESM1_ESM.docx]

**Supplementary table 1.** Viable cell count (Log CFU/mL) of *C. albicans* and *S. mutans* in dual-species biofilms formed on polymethylmethacrylate discs after exposure to test agents for 3 and 15 minutes and untreated control (N = 81).

| **Groups** | **Viable cell count (Log CFU/mL)** | | | | | | | | | | | | | | |
| --- | --- | --- | --- | --- | --- | --- | --- | --- | --- | --- | --- | --- | --- | --- | --- |
|  | ***C. albicans*** | | | | | | |  | ***S. mutans*** | | | | | | |
|  | **3-minute** | | |  | **15-minute** | | |  | **3-minute** | | |  | **15-minute** | | |
|  | **Median** | **Min** | **Max** |  | **Median** | **Min** | **Max** |  | **Median** | **Min** | **Max** |  | **Median** | **Min** | **Max** |
| 2% CHX | 0.00 | 0.00 | 3.56 |  | 0.00 | 0.00 | 3.76 |  | 4.62 | 0.00 | 6.61 |  | 0.00 | 0.00 | 4.31 |
| Compound 1 | 4.62 | 3.00 | 6.02 |  | 0.00 | 0.00 | 0.00 |  | 6.61 | 2.49 | 7.15 |  | 0.00 | 0.00 | 0.00 |
| Polident | 6.26 | 6.10 | 6.79 |  | 0.00 | 0.00 | 4.92 |  | 6.60 | 5.92 | 6.88 |  | 0.00 | 0.00 | 0.00 |
| DW | 6.23 | 6.17 | 6.53 |  | 6.05 | 5.11 | 6.12 |  | 7.14 | 6.98 | 7.25 |  | 6.59 | 6.00 | 7.11 |
| Control | 6.21 | 5.61 | 6.48 |  | 6.21 | 5.61 | 6.48 |  | 6.85 | 6.48 | 7.13 |  | 6.85 | 6.48 | 7.13 |
| Biofilm assays were performed in triplicate in 3 independent experiments (n = 9).  CFU/mL: colony forming unit per milliliter; Min: minimum; Max: maximum; DW: distilled water; CHX: Chlorhexidine gluconate. | | | | | | | | | | | | | | | |
